# Rapid multiplex detection of 10 foodborne pathogens with an up-converting phosphor technology-based 10-channel lateral flow assay

**DOI:** 10.1038/srep21342

**Published:** 2016-02-17

**Authors:** Yong Zhao, Haoran Wang, Pingping Zhang, Chongyun Sun, Xiaochen Wang, Xinrui Wang, Ruifu Yang, Chengbin Wang, Lei Zhou

**Affiliations:** 1 Laboratory of Analytical Microbiology, State Key Laboratory of Pathogen and Biosecurity, Beijing Institute of Microbiology and Epidemiology, Beijing 100071, P. R. China; 2 Beijing Key Laboratory of POCT for Bioemergency and Clinic (No. BZ0329), Beijing 100071, P. R. China; 3 School of Food and Nutrition, Massey University, Palmerston North 4442, New Zealand; 4 Department of Clinical Laboratory, Chinese People’s Liberation Army General Hospital, Beijing 100853, P. R. China; 5 College of Animal Science and Technology, Jilin Agricultural University, Changchun 130118, P. R. China; 6 Institute for Plague Prevention and Control of Hebei Province, Zhangjiakou 075000, P. R. China

## Abstract

The rapid high-throughput detection of foodborne pathogens is essential in controlling food safety. In this study, a 10-channel up-converting phosphor technology-based lateral flow (TC-UPT-LF) assay was established for the rapid and simultaneous detection of 10 epidemic foodborne pathogens. Ten different single-target UPT-LF strips were developed and integrated into one TC-UPT-LF disc with optimization. Without enrichment the TC-UPT-LF assay had a detection sensitivity of 10^4^ CFU mL^−1^ or 10^5^ CFU mL^−1^ for each pathogen, and after sample enrichment it was 10 CFU/0.6 mg. The assay also showed good linearity, allowing quantitative detection, with a linear fitting coefficient of determination (R^2^) of 0.916–0.998. The 10 detection channels did not cross-react, so multiple targets could be specifically detected. When 279 real food samples were tested, the assay was highly consistent (100%) with culture-based methods. The results for 110 food samples artificially contaminated with single or multiple targets showed a high detection rate (≥80%) for most target bacteria. Overall, the TC-UPT-LF assay allows the rapid, quantitative, and simultaneous detection of 10 kinds of foodborne pathogens within 20 min, and is especially suitable for the rapid detection and surveillance of foodborne pathogens in food and water.

Foodborne diseases, which are mainly caused by foodborne pathogens, are a serious health hazard in both developing and developed countries. *Salmonella* spp., *Escherichia coli* O157:H7, *Vibrio parahaemolyticus, V. cholerae* O1, and *V. cholerae* O139 are the most commonly encountered foodborne-disease-related bacterial pathogens^1^,^2^. *Salmonella* spp. are the leading bacterial cause of acute gastrointestinal illness, with nearly 20,000 hospitalizations and 378 deaths per year in the United States^3^. Common *Salmonella* serotypes that can cause foodborne illnesses include *S. enteritidis*, *S. typhi*, *S. paratyphi* A, *S. paratyphi* B, *S. paratyphi* C, and *S. choleraesuis*^4^,^5^. These pathogens can exist (and usually coexist) in various foods, such as raw or undercooked foods (meat, seafood, poultry, etc.) and ready-to-eat products (vegetables, fruits, dairy products, etc.)^1^,^6^,^7^, and once consumed by humans, can cause serious infectious diseases, with high rates of hospitalization and death^8^. The on-site screening and surveillance of food and water for various foodborne pathogens is essential to ensure food safety and minimize the occurrence of foodborne diseases. Therefore, an appropriate technique for detecting multiple food-borne pathogens rapidly and simultaneously must be developed.

The current gold-standard method for detecting foodborne pathogens is the culture-based bacterial isolation and identification, but the procedures are tedious and time-consuming (2–3 days)^9^. Many new methods have emerged for the rapid diagnosis of bacterial infections, including real-time polymerase chain reaction (PCR)^10^,^11^, DNA microarrays^12^,^13^, and enzyme-linked immunosorbent assays (ELISAs)^14^. Real-time PCR and DNA microarrays allow the multiplex detection of bacteria with high sensitivity, but rely on complex sample pre-treatment and expensive instrumentation^15^. ELISAs are a widely used immunological approach that is less complicated and less expensive; however, it is still difficult to realize direct applications in the filed because of the dependence on equipment and professional operation^9^. Therefore, these techniques do not meet the criteria for the urgently needed on-site multiplex detection system for foodborne pathogens.

The lateral flow assay (LFA) is the classical point-of-care bacterial testing technique^16^. Based on the immunoreaction between an antibody and antigen, it can detect pathogens rapidly, simply, and cheaply. The technique is improved when an up-converting phosphor (UCP) particle as the reporter is used^17^, in a process called an ‘UCP-technology-based lateral flow assay’ (UPT-LFA), which allows the quantitative detection of bacteria with higher sensitivity and tolerance of sample interference than other methods. This is based on the unique optical features of the anti-Stokes shift and the stable fluorescence of UCP^18^,^19^,^20^, which provide a solid foundation for the on-site rapid detection of pathogens. Many multiplex UPT-LFAs have been established by coating two or more specific test lanes on one strip with a common UCP or UCPs with different optical spectra as the signal reporters^21^,^22^. Therefore, the results can be analysed with “position encoding” and/or “signal encoding”^23^. However, this technique has several limitations. (1) Position encoding using multiple lanes in one strip can increase the risk of false binding. (2) Signal encoding requires multiple settings of the optical source, filter, and receiver, which increase the complexity and the bulk of the analytical instrument required. In a previous study^24^, we developed a rapid assay to simultaneously detect 10 different antibodies against *Yersinia pestis* with a 10-channel UCP-technology-based lateral flow (TC-UPT-LF) disc. The disc holds 10 detection channels, with one strip in each channel using the same UCP reporter, and thus efficiently reduces or abolishes the risk of nonspecific binding, allowing multiplex detection based on one optical system.

In this study, we developed a TC-UPT-LF assay for the rapid and simultaneous detection of 10 epidemic foodborne pathogens: *E. coli* O157:H7, *S. paratyphi* A, *S. paratyphi* B, *S. paratyphi* C, *S. typhi*, *S. enteritidis*, *S. choleraesuis*, *V. cholera* O1, *V. cholera* O139, and *V. parahaemolyticus*. The performance of the multiplex assay, including its sensitivity, specificity, and precision in quantitative analyses, was comprehensively evaluated in comparison with single-target UPT-LF strip assays. The capacity of the proposed assay to detect multiple target pathogens simultaneously was also tested, and the effect of non-target interference on the assay performance was evaluated. The results obtained with artificially contaminated food samples and real samples demonstrate that the TC-UPT-LF assay can simultaneously detect these 10 target foodborne pathogens in foods with high sensitivity and reliability.

## Results and Discussion

### Establishment of the TC-UPT-LF assay to detect 10 foodborne pathogens

**Figure 1:**
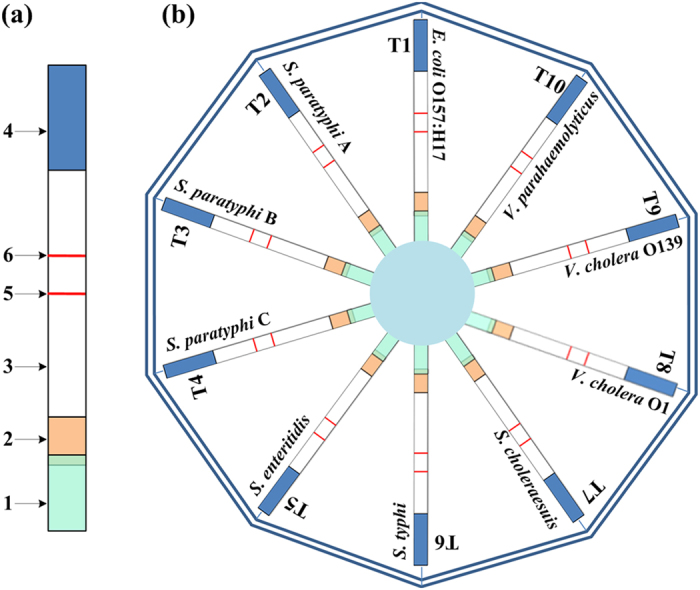
Schematic description of the UPT-LF strip and TC-UPT-LF disc. (**a**) The strip is composed of a sample pad (1), a conjugate pad (2), a nitrocellulose membrane (3), and an absorbent paper (4). During preparation, the conjugate pad was fixed with the UCP–antibody complex, and the membrane was coated with a test lane (5) and a control lane (6). (**b**) The TC-UPT-LF disc holds 10 detection channels (T1 to T10), which contain the appropriate UPT-LF strips for the target bacteria.

To establish the TC-UPT-LF assay, we first developed single-target UPT-LF strips for each of the 10 foodborne pathogens, based on a double-antibody sandwich immunoassay. The optimal sensitivities of the 10 single-target UPT-LF strips (Table 1) were determined using the corresponding optimized sample treatment buffers (see Supplementary Table S1). The 10 single-target UPT-LF strips were then integrated into the TC-UPT-LF disc, which holds 10 channels for strips, as shown in Fig. 1. The strips were overlapped at the sides of the sample pad. Liquid samples could be distributed synchronously and uniformly into the 10 channels through the drainage piece located in the disc center. Because the sample treatment buffers for the strips all differed, an optimized buffer (0.05 M Tris-HCl [pH 8.0] containing 2.5% defatted milk powder and 0.25% SDS) was chosen as the universal sample treatment buffer to facilitate the one-step application of samples to the TC-UPT-LF disc.

### Sensitivity and quantitative linearity of the TC-UPT-LF assay

**Figure 2:**
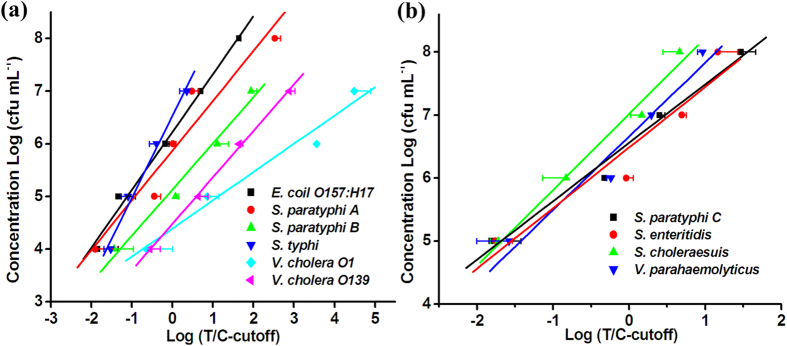
Quantification curves of the target bacteria determined with the TC-UPT-LF assay. The *x*-axis refers to log_10_(T/C – cut-off); the *y*-axis refers to log_10_(concentration of target bacteria [CFU mL^−1^]).

The performance of the TC-UPT-LF assay was evaluated with serial dilutions of pure cultures of the target bacteria. The minimum concentration detected, based on a T/C ratio (signal intensity of T lane/signal intensity of C lane) above the corresponding cut-off value (mean ± 3 standard deviations [SD] of the T/C ratios of the blank controls) was deemed to be the detection sensitivity. The results showed that detection sensitivity of the assay for each target was 10^4^ CFU mL^−1^ or 10^5^ CFU mL^−1^ (depending on the target) (Table 1). The quantitative curve for each target was constructed, with log_10_(T/C - cut-off) on the *x*-axis and log_10_(target bacterial concentration [CFU mL^−1^]) on the *y*-axis (Fig. 2). As these curves show, the assay accurately quantified bacteria across a wide range of concentrations, with R^2^ of 0.916–0.998. The quantification equations for the 10 target species are presented in Table 1.

The detection sensitivity of the new assay for *S. paratyphi* A, *S. paratyphi* B, *S. paratyphi* C, *S. enteritidis*, *S. typhi*, and *V. parahaemolyticus* did not differ markedly from that of the corresponding single-target UPT-LF strip assays. However, the sensitivity of the multiplex assay was 10-fold lower than that of the single-target assays for the other four target bacteria: *E. coli* O157:H17, *S. choleraesuis*, *V. cholera* O1, and *V. cholera* O139. We inferred that this reduction in sensitivity was mainly attributable to the different sample treatment buffers used in the two assays. The sample treatment buffers for the single-target UPT-LF assays were developed to ensure of the optimal performance of the assay for a specific target. However, the universal buffer used for the TC-UPT-LF assay was optimized for use with 10 strips simultaneously, which reduced the sensitivity of the assay for some targets.

### Specificity of the TC-UPT-LF assay

**Figure 3:**
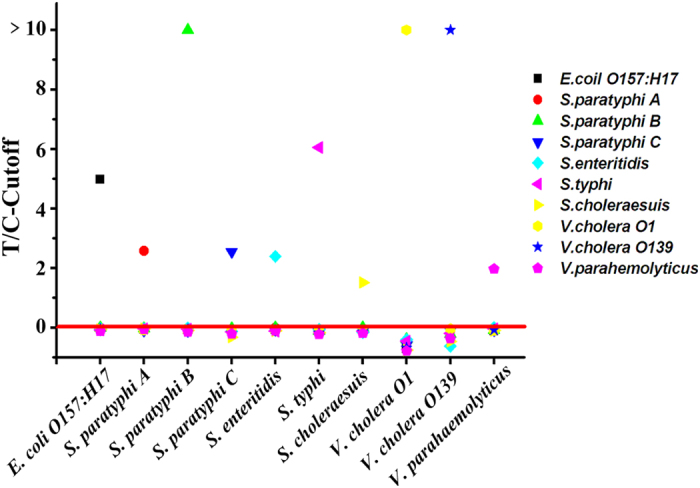
Specificity of each detection channel of the TC-UPT-LF disc. The *x*-axis refers to the 10 detection channels on the disc; the *y*-axis refers to the results (T/C - cut-off) for the corresponding bacterial solutions (1.0 × 10^8^ CFU mL^−1^). Results were deemed positive when T/C - cut-off > 0 and negative when T/C - cut-off ≤0.

The specificity of each detection channel of the TC-UPT-LF assay was assessed individually with high concentrations (1.0 × 10^8^ CFU mL^−1^) of the other nine species of foodborne bacteria. As the results show (Fig. 3), only the target bacterium displayed a strong signal in the corresponding detection channel, with a T/C ratio above the cut-off value, whereas the T/C ratios of the non-target bacteria were all below the cut-off value. This indicates that each detection channel in the disc specifically detected its target, with no cross-reactivity with the other bacteria, confirming the high specificity of the assay. High specificity is essential for the simultaneous detection of the 10 pathogens by the TC-UPT-LF assay.

### Simultaneous multiplex detection of 10 pathogens

Ten different target bacteria were prepared in one solution (at a final concentration of 1.0 × 10^7^ CFU mL^−1^ for each target) to evaluate the capacity of the TC-UPT-LF assay to detect multiple targets simultaneously. All 10 targets were detected simultaneously. Some targets, including *E. coli* O157:H17, *S. enteritidis*, *S. choleraesuis, V. cholera* O139, and *V. parahaemolyticus*, were also correctly quantified (as 10^7^ CFU mL^−1^) with the corresponding quantification curves. The other five pathogens displayed lower T/C ratios and their concentrations were underestimated (10^6^ CFU mL^−1^). This phenomenon was attributed to the blocking effect of the non-target bacteria on the specific immunoreaction between the antibodies and the target bacteria, and should not significantly influence the simultaneous screening of the 10 target bacteria.

**Figure 4:**
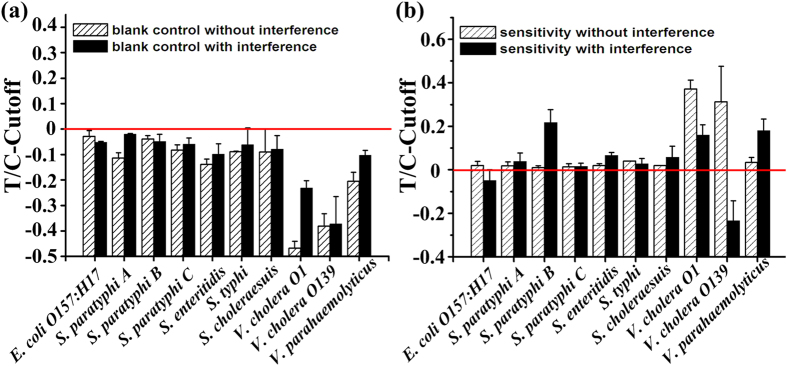
Multiplex detection results with the TC-UPT-LF assay and effects of non-target bacteria on assay performance. (**a**) Effect of interference by non-target bacteria on the specificity (blank controls) of each detection channel. (**b**) Effect of interference by non-target bacteria on the sensitivity of each detection channel.

Furthermore, we evaluated the interference by excessive non-target pathogens on the detection specificity and sensitivity of the assay. Serial concentrations of the target bacteria (from 0 CFU mL^−1^ to 1.0 × 10^8^ CFU mL^−1^), mixed with all the other nine pathogens (each at a final concentrations of 1.0 × 10^7^ CFU mL^−1^), were prepared and tested with the TC-UPT-LF assay. Although the T/C ratios of the target bacteria at 0 CFU mL^−1^ (blank control, combined with the non-target bacteria) were elevated to different extents (Fig. 4a), all were well below the cut-off value for the corresponding channel, which demonstrates the high specificity of each channel in the multiplex assay.

As shown in Fig. 4b, the detection sensitivity of the assay was maintained at the same level for most of the 10 target bacteria (except *E. coli* O157:H17 and *V. cholera* O139), even during interference with high concentrations of non-target bacteria. However, the T/C ratios for *E. coli* O157:H17 and *V. cholera* O139 at the concentration of 1.0 × 10^4^ CFU mL^−1^ were reduced to below the cut-off value for the corresponding channel. The detection sensitivity of the assay for these two targets increased slightly from 1.0 × 10^4^ CFU mL^−1^ to 1.0 × 10^5^ CFU mL^−1^. We attributed this to the blocking effects of high concentrations of non-target bacteria (which also affected the accuracy of quantitation). However, with the excellent specificity of the assay, the effects of interference by non-target bacteria on its sensitivity and quantitative accuracy were acceptable. Overall, these results suggest that the assay performs well in the simultaneous detection of these 10 bacteria.

### Application of the TC-UPT-LF assay to food samples

We tested 279 food samples, including dairy products, marine products, beverages, snacks, and meats, with both the TC-UPT-LF assay and the standard method of bacterial isolation and culture. The results showed an excellent coincidence rate of 100% between the two methods, which both showed that all samples were free of the 10 targeted foodborne pathogens.

Because the food samples collected in the field were all negative for pathogens, 100 single-target food samples were prepared by artificially contaminating them with about 10 CFU of a specific bacterium. These single-target-infected samples were enriched and tested with the TC-UPT-LF assay, as described above. The assay specifically and accurately identified 88% (88/100) of the pathogens in the samples (Table 2). The other 12 samples were all misidentified as negative, which may be attributable to a failure of the contamination by or enrichment of the trace amounts of bacteria in the food samples. The assay also showed a high success rate in detecting each target bacterium. The targets *E. coli* O157:H17, *S. paratyphi* B, and *V. parahaemolyticus* in the samples were all accurately identified, with detection rates of 100% (10/10). The other targets, *S. paratyphi* A, *S. paratyphi* C, *S. typhi*, *S. enteritidis*, *V. cholera* O1, and *V. cholera* O139, were identified with detection rates of ≥80% (8/10). Only the detection rate of *S. choleraesuis* was 70% (7/10). Overall, these results demonstrate the relatively high accuracy and specificity of the TC-UPT-LF assay in detecting these 10 pathogens in food matrices.

Ten food samples contaminated with all 10 pathogens simultaneously were also prepared and tested with the assay. As shown in Table 3, only four samples were correctively identified as contaminated with all 10 target bacteria. The other samples were shown to contain at least seven different target pathogens. The detection rates for *S. paratyphi* A, *S. choleraesuis*, *V. cholera* O1, and *V. cholera* O139 in the 10-target samples were lower than those in the single-target samples, especially for *V. cholera* O1, the detection rate of which decreased from 90% to 60% in the multiply contaminated samples (Table 2). We inferred that the interactions between the 10 pathogens in the same broth were complex, and may have inhibited the growth of particular bacteria, reducing the detection rate of the assay. However, most target bacteria were still identified with high detection rates (≥80%), confirming the capacity of the assay to detect multiple target pathogens simultaneously in food samples.

## Conclusion

In this study, a TC-UPT-LF assay was developed to allow the one-step quantitative detection of 10 kinds of foodborne pathogens within 20 min. Compared with single-target UPT-LF strips, the multiplex assay showed similar quantitative accuracy, sensitivity, and specificity in the simultaneous detection of the 10 pathogens. After sample enrichment, the assay identified the target bacteria in various food matrices, with a detection sensitivity of around 10 CFU/0.6 mg. It also performed well despite interference by numerous non-target organisms in each channel of the TC-UPT-LF disc, allowing the reliable simultaneous detection of multiple pathogens. Because it is rapid, easy to perform, inexpensive, and permits high throughput, the TC-UPT-LF disc assay is a promising tool for the rapid detection and surveillance of foodborne pathogens in food or water.

## Methods

### Ethics statement

Monoclonal antibodies (mAbs) against the target bacteria used in this study were prepared. Eight-week-old female Balb/c mice were obtained from the Laboratory Animal Research Center, Academy of Military Medical Sciences (China). Mouse acquisition was approved by the Ministry of Health in the General Logistics Department of the Chinese People’s Liberation Army. All experiments were performed in accordance with the Guidelines for the Welfare and Ethics of Laboratory Animals of China. All experimental protocols were approved by the Committee of the Welfare and Ethics of Laboratory Animals, Beijing Institute of Microbiology and Epidemiology (Beijing, China).

### Reagents and materials

UCP (NaYF_4_:Yb^3+^/Er^3+^), with a diameter of about 50 nm, was obtained from Shanghai Kerune Phosphor Technology Co. Ltd (Shanghai, China). The peaks of its excitation and emission spectra were 980 nm and 541.5 nm, respectively. Nitrocellulose membrane (SHF 1350225) and glass fibre (GFCP20300) were purchased from Millipore Corp. (Bedford, MA, USA). Papers (nos 470 and 903) used to make the sample pad and absorbent pad were obtained from Schleicher and Schuell, Inc. (Keene, NH, USA). The laminated cards and plastic cartridges of the TC-UPT-LF disc were designed by our group and manufactured by Shanghai Liangxin Biotechnology Co. (Shanghai, China) and Shenzhen Jincanhua Industry Co. (Shenzhen, China), respectively.

The reagents, including tryptone, yeast extract powder, NaCl, tris(hydroxymethyl)aminomethane, HCl, Na_2_HPO_4_, KH_2_PO_4_, and SDS were all of analytical grade and purchased from Sigma-Aldrich (St. Louis, MO, USA). Defatted milk powder was obtained from a local supermarket.

### Bacterial culture

*Vibrio cholera* O1 and *V. cholera* O139 were obtained from the Chinese Centers for Disease Control and Prevention (Beijing, China); the other eight target pathogens are maintained in our laboratory. Alkaline peptone water (peptone 20 g L^−1^, Na_2_CO_3_.12H_2_O 0.2 g L^−1^, NaCl 5 g L^−1^, KNO_3_ 0.1 g L^−1^ [pH 8.4]) was used to culture *V. cholera* O1 and *V. cholera* O139, and LBS broth (tryptone 20 g L^−1^, yeast extract powder 10 g L^−1^, NaCl 10 g L^−1^ [pH 7.2]) was used for the other eight bacteria. All bacteria were grown overnight at 37 °C with shaking at 200 rpm.

### TC-UPT-LF biosensor

The TC-UPT-LF biosensor was designed and fabricated by our laboratory and the Shanghai Institute of Optics and Fine Mechanics, Chinese Academy of Sciences (Shanghai, China). Unlike the single-channel UPT biosensor^25^, it contains a high-precision two-dimensional disc platform that allows both linear and rotary motion of the disc and channel shifting. Therefore, it can capture signals from the 10 channels of the disc consecutively.

### Establishment of the TC-UPT-LF assay

The UPT-LF strips used in the study were developed based on a double-antibody sandwich immunoassay. Initially, the conjugate pad was fixed with the UCP–mAb conjugate (1 mg mL^−1^, 30 μL cm^−1^). The nitrocellulose membrane was coated with the corresponding mAb (2 mg mL^−1^, 1 μL cm^−1^) directed against the target bacterium in the test lane (T lane) and the goat anti-mouse IgG antibody (2 mg mL^−1^, 1 μL cm^−1^) in the control lane (C lane). The sample pad, the conjugate pad, the nitrocellulose membrane, and the absorbent paper were then assembled as shown in Fig. 1.

The TC-UPT-LF disc holds 10 channels, which can contain 10 different single-target UPT-LF strips, developed as described above. The strips in the disc are overlapped at the side of sample pad. Liquid samples can be distributed synchronously and uniformly into the 10 channels through the drainage piece (glass fiber) located in the disc center.

Before detection, a bacterial solution and the optimized universal sample treatment buffer (0.05 M Tris-HCl [pH 8.0] containing 2.5% defatted milk powder and 0.25% SDS) were thoroughly mixed in a ratio of 1:9. An aliquot (1.4 mL) of the mixture was then added to the disc. After 15 min, the signals from the disc were scanned via the TC-UPT-LF biosensor. Signal reading and reporting were completed within 2 min. The peak areas of the test lane and the control lane are referred to as the ‘T value’ and the ‘C value’, respectively. The T/C ratio was then calculated and regarded as the result. Samples with T/C ratios higher than the cut-off value (mean ± 3SD of the T/C ratio for the blanks) were deemed to be positive and those with T/C ratios lower than the cut-off value were deemed to be negative. The whole operation was completed within 20 min, from sample processing to result.

### Evaluation of sensitivity, quantification linearity, and specificity of the TC-UPT-LF assay

Bacterial cultures were harvested and stored in sterile saline (0.85% salt solution) at a final concentration of 1.0 × 10^9^ CFU mL^−1^, determined with the plate count method. The standard solutions of each target pathogen were diluted with phosphate buffer (0.02 M Na_2_HPO_4_, 0.01 M KH_2_PO_4_ [pH 7.2]), ranging from 1.0 × 10^3^ CFU mL^−1^ to 1.0 × 10^8^ CFU mL^−1^. All the standard bacterial solutions and the blank control (phosphate buffer) were tested with the TC-UPT-LF assay in triplicate. As a comparison, these solutions were also tested with the corresponding single-target UPT-LF strips. The minimum concentration of target pathogen that could be positively detected was deemed to be the detection sensitivity of the assay. The quantification curve for each target bacterium was constructed with log_10_(T/C - cut-off) on the *x*-axis and log_10_(concentration of bacterial solution [CFU mL^−1^]) on the *y*-axis, with OriginPro 8.0. The linear fitting coefficient of determination (R^2^) was calculated to evaluate the quantitative linearity. The specificity of each detection channel of the TC-UPT-LF disc was assessed individually with the other nine kinds of foodborne bacteria at concentrations of 1.0 × 10^8^ CFU mL^−1^, as described above.

### Multiplex detection of the 10 targets with the TC-UPT-LF assay

Ten different target bacteria were prepared in one solution (at a final concentration of 1.0 × 10^7^ CFU mL^−1^ for each target) to evaluate the capacity of the TC-UPT-LF assay to detect multiple targets simultaneously. The effects of non-target bacteria on the sensitivity and specificity of the multiplex assay were also assessed with serial concentrations of the target bacterial solution (from 0 CFU mL^−1^ to 1.0 × 10^8^ CFU mL^−1^), mixed with the other nine pathogens (each at a final concentration of 1.0 × 10^7^ CFU mL^−1^).

### Detection of target bacteria in food samples with the TC-UPT-LF assay

We obtained 279 food samples from the Shanghai Entry-Exit Inspection and Quarantine Bureau (Shanghai, China), including dairy products, marine products, beverages, snacks, and meat. Before testing, the food samples were aseptically weighed and homogenized by hand, and cultured in 5 mL of LBS broth (using 700 μL of liquid samples, 0.6 mg of solid samples) for around 14 h, and were then tested as described above. Standard methods of bacterial culture, isolation, and identification were used.

### Detection of target bacteria in spiked food samples with the TC-UPT-LF assay

Food samples (from the negative samples among the real food samples) artificially contaminated with the 10 pathogens were also prepared, including 100 single-target samples containing one of the 10 target bacteria and 10 samples contaminated with all 10 target bacteria. Because food samples might be contaminated with trace amounts of pathogens (<100 CFU g^−1^), which are unlikely to be detected directly^22^,^23^, the samples were spiked with around 10 CFU of bacteria (10 μL of bacterial solution at a concentration of 1000 CFU mL^−1^). These samples were used to inoculate 5 mL of LBS broth, which was incubated for around 14 h, and then tested with the TC-UPT-LF assay, as described above.

## Additional Information

**How to cite this article**: Zhao, Y. *et al.* Rapid multiplex detection of 10 foodborne pathogens with an up-converting phosphor technology-based 10-channel lateral flow assay. *Sci. Rep.* **6**, 21342; doi: 10.1038/srep21342 (2016).

## Supplementary Material

Supplementary Information

## Figures and Tables

**Table 1:** Detection sensitivity and quantitation capacity of the TC-UPT-LF assay for 10 pathogens.

Target pathogen	Detection sensitivity (CFU mL^−1^)		quantitative equations of theTC-UPT-LF (R^2^)
	Single-targetUPT-LF assay	TC-UPT-LFassay	
*E. coil* O157:H17	1.0 × 10^3^	1.0 × 10^4^	*y* = 1.099*x* + 6.225 (0.990)
*S. paratyphi* A	1.0 × 10^4^	1.0 × 10^4^	*y* = 0.945*x* + 5.872 (0.926)
*S. paratyphi* B	1.0 × 10^4^	1.0 × 10^4^	*y* = 0.878*x* + 5.132 (0.982)
*S. paratyphi* C	1.0 × 10^5^	1.0 × 10^5^	*y* = 0.928*x* + 6.561 (0.982)
*S. enteritidis*	1.0 × 10^5^	1.0 × 10^5^	*y* = 0.962*x* + 6.486 (0.916)
*S. typhi*	1.0 × 10^3^	1.0 × 10^4^	*y* = 1.559*x* + 6.532 (0.986)
*S. choleraesuis*	1.0 × 10^4^	1.0 × 10^5^	*y* = 1.206*x* + 7.019 (0.983)
*V. cholera* O1	1.0 × 10^3^	1.0 × 10^4^	*y* = 0.537*x* + 4.392 (0.969)
*V. cholera* O139	1.0 × 10^3^	1.0 × 10^4^	*y* = 0.883*x* + 4.480 (0.998)
*V. parahaemolyticus*	1.0 × 10^5^	1.0 × 10^5^	*y* = 1.169*x* + 6.663 (0.955)

“*x*” refers to log_10_(T/C - cut-off); “*y*” refers to log_10_(concentration of target bacteria [CFU mL^−1^]).

**Table 2:** Detection rates of the TC-UPT-LF assay for single-target-containing and multiple-target-containing food samples.

Target pathogens	Detection rate by the TC-UPT-LF	
	Single-target samples	Multiple-target samples
*E. coil* O157:H17	100% (10/10)	100% (10/10)
*S. paratyphi* A	90% (9/10)	80% (8/10)
*S. paratyphi* B	100% (10/10)	100% (10/10)
*S. paratyphi* C	80% (8/10)	100% (10/10)
*S. enteritidis*	80% (8/10)	100% (10/10)
*S. typhi*	80% (8/10)	90% (9/10)
*S. choleraesuis*	70% (7/10)	60% (6/10)
*V. cholera* O1	90% (9/10)	60% (6/10)
*V. cholera* O139	90% (9/10)	80% (8/10)
*V. parahaemolyticus*	100% (10/10)	100% (10/10)
Total	88% (88/100)	–

**Table 3:** Results of the TC-UPT-LF assay for food samples contaminated with the 10 target bacteria.

10-targetsamples ID No.	Results of the ten channels in the disc										Positivenumber
	T1	T2	T3	T4	T5	T6	T7	T8	T9	T10	
1	P	N	P	P	P	P	P	N	P	P	8
2	P	P	P	P	P	P	P	N	P	P	9
3	P	N	P	P	N	P	N	P	P	P	7
4	P	P	P	P	P	P	N	N	N	P	7
5	P	P	P	P	P	P	N	N	N	P	7
6	P	P	P	P	P	P	P	P	P	P	10
7	P	P	P	P	P	P	P	P	P	P	10
8	P	P	P	P	P	P	N	P	P	P	9
9	P	P	P	P	P	P	P	P	P	P	10
10	P	P	P	P	P	P	P	P	P	P	10

“P” refers to positive detection; “N” refers to negative detection.

## References

[b1] Law J. W., Ab Mutalib N. S., Chan K. G. & Lee L. H. Rapid methods for the detection of foodborne bacterial pathogens: principles, applications, advantages and limitations. Frontiers in microbiology 5, 770 (2014). 25628612 10.3389/fmicb.2014.00770 PMC4290631

[b2] Chao G., Zhou X., Jiao X., Qian X. & Xu L. Prevalence and antimicrobial resistance of foodborne pathogens isolated from food products in China. Foodborne pathogens and disease 4, 277–284 (2007). 17883311 10.1089/fpd.2007.0088

[b3] Scallan E. *et al.* Foodborne illness acquired in the United States–major pathogens. Emerging infectious diseases 17, 7–15 (2011). 21192848 10.3201/eid1701.P11101 PMC3375761

[b4] Crump J. A. & Mintz E. D. Global trends in typhoid and paratyphoid Fever. Clinical infectious diseases: an official publication of the Infectious Diseases Society of America 50, 241–246 (2010). 20014951 10.1086/649541 PMC2798017

[b5] Kim S. *et al.* Multiplex PCR-based method for identification of common clinical serotypes of Salmonella enterica subsp. enterica. Journal of clinical microbiology 44, 3608–3615 (2006). 16943358 10.1128/JCM.00701-06 PMC1594780

[b6] Vinothkumar K., Bhardwaj A. K., Ramamurthy T. & Niyogi S. K. Triplex PCR assay for the rapid identification of 3 major Vibrio species, Vibrio cholerae, Vibrio parahaemolyticus, and Vibrio fluvialis. Diagn Micr Infec Dis 76, 526–528 (2013). 10.1016/j.diagmicrobio.2013.04.005 23706502

[b7] Oliver S. P., Jayarao B. M. & Almeida R. A. Foodborne pathogens in milk and the dairy farm environment: food safety and public health implications. Foodborne pathogens and disease 2, 115–129 (2005). 15992306 10.1089/fpd.2005.2.115

[b8] Newell D. G. *et al.* Food-borne diseases - the challenges of 20 years ago still persist while new ones continue to emerge. International journal of food microbiology 139 Suppl 1, S3–15 (2010). 20153070 10.1016/j.ijfoodmicro.2010.01.021 PMC7132498

[b9] Zhao X., Lin C. W., Wang J. & Oh D. H. Advances in rapid detection methods for foodborne pathogens. Journal of microbiology and biotechnology 24, 297–312 (2014). 24375418 10.4014/jmb.1310.10013

[b10] Kawasaki S. *et al.* Evaluation of a multiplex PCR system for simultaneous detection of Salmonella spp., Listeria monocytogenes, and *Escherichia coli* O157:H7 in foods and in food subjected to freezing. Foodborne Pathog Dis 6, 81–89 (2009). 18991547 10.1089/fpd.2008.0153

[b11] Kawasaki S. *et al.* Multiplex real-time polymerase chain reaction assay for simultaneous detection and quantification of Salmonella species, Listeria monocytogenes, and *Escherichia coli* O157:H7 in ground pork samples. Foodborne pathogens and disease 7, 549–554 (2010). 20132032 10.1089/fpd.2009.0465

[b12] Suo B. *et al.* Development of an oligonucleotide-based microarray to detect multiple foodborne pathogens. Molecular and cellular probes 24, 77–86 (2010). 19833198 10.1016/j.mcp.2009.10.005

[b13] Huang A. *et al.* High-throughput detection of food-borne pathogenic bacteria using oligonucleotide microarray with quantum dots as fluorescent labels. International journal of food microbiology 185, 27–32 (2014). 24927399 10.1016/j.ijfoodmicro.2014.05.012

[b14] Shen Z. *et al.* A novel enzyme-linked immunosorbent assay for detection of *Escherichia coli* O157:H7 using immunomagnetic and beacon gold nanoparticles. Gut pathogens 6, 14 (2014). 24864164 10.1186/1757-4749-6-14 PMC4033681

[b15] Buckwalter S. P. *et al.* Inhibition controls for qualitative real-time PCR assays: are they necessary for all specimen matrices? J Clin Microbiol 52, 2139–2143 (2014). 24740078 10.1128/JCM.03389-13 PMC4042775

[b16] Cheung S. F., Cheng S. K. & Kamei D. T. Paper-Based Systems for Point-of-Care Biosensing. Journal of laboratory automation 20, 316–333 (2015). 25787805 10.1177/2211068215577197

[b17] Hampl J. *et al.* Upconverting phosphor reporters in immunochromatographic assays. Analytical biochemistry 288, 176–187 (2001). 11152588 10.1006/abio.2000.4902

[b18] Corstjens P. L. *et al.* Tools for diagnosis, monitoring and screening of Schistosoma infections utilizing lateral-flow based assays and upconverting phosphor labels. Parasitology 141, 1841–1855 (2014). 24932595 10.1017/S0031182014000626 PMC4265670

[b19] Zhang P. *et al.* Evaluation of up-converting phosphor technology-based lateral flow strips for rapid detection of Bacillus anthracis Spore, Brucella spp., and Yersinia pestis. PloS one 9, e105305 (2014). 25144726 10.1371/journal.pone.0105305 PMC4140738

[b20] Yan Z. *et al.* Rapid quantitative detection of Yersinia pestis by lateral-flow immunoassay and up-converting phosphor technology-based biosensor. Sensors and Actuators B: Chemical 119, 656–663 (2006). 10.1016/j.snb.2006.01.029 PMC7125792 32288237

[b21] Corstjens P. L. *et al.* Rapid assay format for multiplex detection of humoral immune responses to infectious disease pathogens (HIV, HCV, and TB). Ann Ny Acad Sci 1098, 437–445 (2007). 17435148 10.1196/annals.1384.016

[b22] Wu S., Duan N., Shi Z., Fang C. & Wang Z. Simultaneous aptasensor for multiplex pathogenic bacteria detection based on multicolor upconversion nanoparticles labels. Analytical chemistry 86, 3100–3107 (2014). 24568625 10.1021/ac404205c

[b23] Roda A. *et al.* Recent developments in rapid multiplexed bioanalytical methods for foodborne pathogenic bacteria detection. Microchim Acta 178, 7–28 (2012).

[b24] Hong W. Y. *et al.* Development of an up-converting phosphor technology-based 10-channel lateral flow assay for profiling antibodies against Yersinia pestis. J Microbiol Meth 83, 133–140 (2010). 10.1016/j.mimet.2010.08.005 20801166

[b25] Yan Z. Q. *et al.* Rapid quantitative detection of Yersinia pestis by lateral-flow immunoassay and up-converting phosphor technology-based biosensor. Sensor Actuat B-Chem 119, 656–663 (2006). 10.1016/j.snb.2006.01.029 PMC7125792 32288237

