# Supplementary material for: Rapid multiplex detection of 10 foodborne pathogens with an up-converting phosphor technology-based 10-channel lateral flow assay
Source: Sci Rep. 2016 Feb 17;6:21342. doi: 10.1038/srep21342 (PMC4756364; doi:10.1038/srep21342)
Supplement: Supplementary Information [file srep21342-s1.pdf]

Supplementary Information

Rapid multiplex detection of 10 foodborne pathogens with an up-converting phosphor technology-based 10-channel lateral flow assay

Yong Zhao[1,2,†], Haoran Wang[3,1,†], Pingping Zhang[1,2], Chongyun Sun[1,2,4], Xiaochen Wang[1,2,5], Xinrui Wang[1,2,6], Ruifu Yang[1,2], Chengbin Wang[4,*], Lei Zhou[1,2,*]

[1]Laboratory of Analytical Microbiology, State Key Laboratory of Pathogen and Biosecurity, Beijing Institute of Microbiology and Epidemiology, Beijing 100071, P. R. China

[2]Beijing Key Laboratory of POCT for Bioemergency and Clinic (No. BZ0329), Beijing 100071, P. R. China

[3]School of Food and Nutrition, Massey University, Palmerston North 4442, New Zealand

[4]Department of Clinical Laboratory, Chinese People's Liberation Army General Hospital, Beijing 100853, P. R. China

[5]College of Animal Science and Technology, Jilin Agricultural University, Changchun 130118, P. R. China

[6]Institute for Plague Prevention and Control of Hebei Province, Zhangjiakou 075000, P. R. China

[†]YZ and HW contributed equally to this work.

*Corresponding authors:

Lei Zhou, Tel: +86-10-66948562; E-mail: ammszhoulei@aliyun.com

Chengbin Wang, Tel: +86-10-66937391; E-mail: wangcb301@126.com

Supplementary Table S1. Sample treatment buffers used in the single-target UPT-LF assays and the TC-UPT-LF assay.

**Treatment buffers**	**Buffer compositions**		
E. coil O157:H17	0.05 M Tris-HCl (pH 8.0)	2.5% defatted milk powder	0.25 M NaCl
S. paratyphi A	0.05 M Tris-HCl (pH 8.0)	2.5% defatted milk powder	0.25% SDS
S. paratyphi B	0.05 M Tris-HCl (pH 8.0)	2.5% defatted milk powder	0.25% SDS
S. paratyphi C	0.05 M Tris-HCl (pH 8.0)	2.5% defatted milk powder	0.25% SDS
S. enteritidis	0.05 M Tris-HCl (pH 8.0)	2.5% defatted milk powder	0.1% SDS
S. typhi	0.05 M Tris-HCl (pH 8.0)	2.5% defatted milk powder	0.5 M NaCl
S. choleraesuis	0.05 M Tris-HCl (pH 8.0)	2.5% defatted milk powder	0.25 M NaCl
V. cholera O1	0.05 M Tris-HCl (pH 8.0)	3.5% defatted milk powder	0.5 M NaCl
V. cholera O139	0.05 M Tris-HCl (pH 8.0)	3.5% defatted milk powder	0.5 M NaCl
V. parahaemolyticus	0.05 M Tris-HCl (pH 8.0)	2.5% defatted milk powder	0.1 % SDS
The universal buffer	0.05 M Tris-HCl (pH 8.0)	2.5% defatted milk powder	0.25% SDS
